# A multi-path 2.5 dimensional convolutional neural network system for segmenting stroke lesions in brain MRI images

**DOI:** 10.1016/j.nicl.2019.102118

**Published:** 2019-12-09

**Authors:** Yunzhe Xue, Fadi G. Farhat, Olga Boukrina, A.M. Barrett, Jeffrey R. Binder, Usman W. Roshan, William W. Graves

**Affiliations:** a Department of Computer Science, New Jersey Institute of Technology, Newark, NJ 07102, USA; b Stroke Rehabilitation Research, Kessler Foundation, West Orange, NJ, USA; c Department of Physical Medicine and Rehabilitation, Rutgers – New Jersey Medical School, Newark, NJ, USA; d Department of Neurology, Medical College of Wisconsin, Milwaukee, WI, USA; e Department of Psychology, Rutgers University – Newark, Newark, NJ, USA

**Keywords:** MRI, Convolutional, Neural network, Deep learning, Stroke, Neuropsychology

## Abstract

Automatic identification of brain lesions from magnetic resonance imaging (MRI) scans of stroke survivors would be a useful aid in patient diagnosis and treatment planning. It would also greatly facilitate the study of brain-behavior relationships by eliminating the laborious step of having a human expert manually segment the lesion on each brain scan. We propose a multi-modal multi-path convolutional neural network system for automating stroke lesion segmentation. Our system has nine end-to-end UNets that take as input 2-dimensional (2D) slices and examines all three planes with three different normalizations. Outputs from these nine total paths are concatenated into a 3D volume that is then passed to a 3D convolutional neural network to output a final lesion mask. We trained and tested our method on datasets from three sources: Medical College of Wisconsin (MCW), Kessler Foundation (KF), and the publicly available Anatomical Tracings of Lesions After Stroke (ATLAS) dataset. To promote wide applicability, lesions were included from both subacute (1 to 5 weeks) and chronic ( >  3 months) phases post stroke, and were of both hemorrhagic and ischemic etiology. Cross-study validation results (with independent training and validation datasets) were obtained to compare with previous methods based on naive Bayes, random forests, and three recently published convolutional neural networks. Model performance was quantified in terms of the Dice coefficient, a measure of spatial overlap between the model-identified lesion and the human expert-identified lesion, where 0 is no overlap and 1 is complete overlap. Training on the KF and MCW images and testing on the ATLAS images yielded a mean Dice coefficient of 0.54. This was reliably better than the next best previous model, UNet, at 0.47. Reversing the train and test datasets yields a mean Dice of 0.47 on KF and MCW images, whereas the next best UNet reaches 0.45. With all three datasets combined, the current system compared to previous methods also attained a reliably higher cross-validation accuracy. It also achieved high Dice values for many smaller lesions that existing methods have difficulty identifying. Overall, our system is a clear improvement over previous methods for automating stroke lesion segmentation, bringing us an important step closer to the inter-rater accuracy level of human experts.

## Highlights

• Time and effort for hand-segmenting lesions bottlenecks brain lesion-deficit studies.
• We provide a fully-automated tool for stroke brain lesion segmentation.
• This tool significantly outperforms existing techniques, including on small lesions.
• Freely available tool should accelerate progress in brain lesion-deficit studies.

## 1 Introduction

Neuropsychological studies of brain lesion-deficit relationships are an indispensable means of determining what brain areas are critical for carrying out particular functions. This contrasts with functional brain imaging techniques such as functional magnetic resonance imaging (fMRI). While fMRI is extremely popular and useful, it cannot make strong claims about what brain areas are necessary for the functions being investigated. A major impediment to progress in brain lesion-deficit studies, however, is the labor-intensive and ultimately subjective step of having an expert manually segment brain lesions from MRI scans.

This has been highlighted in previous studies comparing inter-rater variability and speed of human compared to automatic lesion identification. Fiez et al. (2000) report a 67% ( ±  7%) agreement in overlapping voxels between two expert raters across ten subjects. More recently, other groups have reported an inter-rater overlap of 0.73  ±  0.2 between experts performing manual lesion segmentation for the Anatomical Tracings of Lesions After Stroke (ATLAS) database (Liew et al., 2018). When brain lesion segmentation is performed exclusively by experienced neuroradiologists, median inter-rater agreement has been shown to be as high as 0.78 (Neumann et al., 2009). However, their involvement of only a small number of patients (N = 14) and their use of lower-resolution scans (6.5 mm slices rather than the typical 1 mm slices used in research) suggests that an inter-rater agreement of 0.78 may be inflated relative to the 0.67 to 0.73 range that seems typical for research studies.

Aside from concerns with inter-rater reliability, manually segmenting lesions is also time consuming, often taking between 4.8 to 9.6 hours. Methods developed for automating this process, however, can segment lesions in roughly a minute (Wilke et al., 2011). However, manual lesion segmentation remains the method of choice, presumably due to the relatively poor accuracy of available automated methods (Ito, Kim, Liew, 2019, Wilke, de Haan, Juenger, Karnath, 2011). Clearly what is needed is a fast, automated method for brain lesion segmentation with a better accuracy than currently available methods.

Automated statistical inference methods for segmentation of medical images have been proposed in previous work (Aslan, Shalaby, Abdelmunim, Farag, 2013, Aslan, Shalaby, Farag, 2013, Farag, El-Baz, Gimel’farb, 2006, Soliman, Khalifa, Elnakib, El-Ghar, Dunlap, Wang, Gimel’farb, Keynton, El-Baz, 2016). These are mainly based on modeling Gaussian distributions on the data. They can be easier to train than sophisticated deep learning ones. And since they are generative, they can be used for simulation. Indeed, identifying lesions in brain MRI images is a key problem in medical imaging (Akkus, Galimzianova, Hoogi, Rubin, Erickson, 2017, Bernal, Kushibar, Asfaw, Valverde, Oliver, Martí, Lladó, 2019). Previous studies have examined the use of standard machine learning classifiers (Griffis, Allendorfer, Szaflarski, 2016, Maier, Schröder, Forkert, Martinetz, Handels, 2015, Pustina, Coslett, Turkeltaub, Tustison, Schwartz, Avants, 2016) and convolutional neural networks (CNN) (Guerrero, Qin, Oktay, Bowles, Chen, Joules, Wolz, Valdés-Hernández, Dickie, Wardlaw, et al., 2018, He, Zhang, Ren, Sun, 2016, Kamnitsas, Ledig, Newcombe, Simpson, Kane, Menon, Rueckert, Glocker, 2017, Ronneberger, Fischer, Brox, 2015) for solving the problem of automating lesion segmentation. Machine learning methods like random forests tend to perform competitively (Maier et al., 2015) but fare below convolutional neural networks (Rachmadi et al., 2017).

The first convolutional UNet (Ronneberger et al., 2015) and subsequent models such as UResNet (Guerrero et al., 2018) take as input 2D slices of the MRI image in a single orientation. They predict the lesion for each slice separately and then combine the predictions into a volume. This approach has limited accuracy because it does not consider the other two planes in the image volume. Without some method, such as a post-processing mechanism, for considering views from other orientations, models such as this will be inherently limited by how well a lesion can be detected in a single orientation view. For example, a wide and flat lesion might be readily distinguishable from healthy tissue in an axial but not coronal view. Indeed, a lesion that is more visible in sagittal and coronal views than in the axial view is shown in Fig. 13.

To address this limitation, CNN systems have been introduced that can accommodate multiple 2D slice orientations. The dual-path CNN, DeepMedic (Kamnitsas et al., 2017), while not considering multiple 2D orientations, does have two pathways. One is for high and one is for low resolution slices. Lyksborg et al. (2015) use a three-path network, one for each of the canonical axial, sagittal and coronal views. Indeed, multi-path systems with up to eight different network paths have been explored previously (de Brebisson and Montana, 2015). Adding paths, however, comes with a cost of having to fit many additional parameters for each path. Fitting these additional parameters leads to an increased risk of over-fitting, as has been reported for multi-path systems (Bernal et al., 2019).

Multi-path systems must also combine the predictions from each path into a final output. One approach to combining path predictions is a simple majority vote. This was the approach used by Lyksborg et al. (2015). However, this approach risks ignoring important but less frequently represented information, as the outputs from different paths are combined into a final voxel prediction by a simple majority vote. Also, the goal of their network was to segment tumors, where the pathology may present a somewhat different problem than stroke. Indeed, in the current work we show that majority vote performs less well on stroke lesion segmentation than a more inclusive 3D convolutional approach to combining outputs across paths.

We address shortfalls in previous approaches by proposing a novel nine-path system, where each path contains a custom U-Net to accommodate multiple MRI modalities or views, depending on the use case. For example, having both T1 and fluid-attentuated inversion recovery (FLAIR) modalities could be useful for segmenting subacute strokes that have occurred within, say, the last 5 weeks. For more chronic strokes having occurred more than 6 months previous, multiple T1 views might be more useful than combining with FLAIR. This possibility is tested in Table 1 below. Our system considers three different normalizations of the images along each of the three axial, sagittal and coronal views. Our custom U-Net is weak on its own but powerful as a component of our multi-path system. This makes sense in the context of ensemble learning where weak learners can perform better in an ensemble (Freund and Schapire, 1997). We also use a 3D convolutional kernel to merge 2D outputs from each path and show that it gives a better accuracy than majority vote. It is because of this combination of 2D and 3D approaches that we refer to our system as 2.5D.

**Table 1:** Mean Dice coefficients of our method on T1 vs. T1+FLAIR images on Kessler+MCW. Also shown are Wilcoxon rank test p-values and average lesion size of images in the combined and individual datasets.

Data	T1	T1+FLAIR	Wilcoxon rank test p-value	Average lesion size (in pixels)
KF+MCW	0.59	0.63	0.2	58,388
KF	0.47	0.58	0.004*	34,054
MCW	0.74	0.68	0.002*	88,804

More generally, a major motivation for the current study was our experience with existing tools that report performing well within their own cross-validation samples (Griffis, Allendorfer, Szaflarski, 2016, Pustina, Coslett, Turkeltaub, Tustison, Schwartz, Avants, 2016), yet perform poorly when applied to scans acquired at a different site. These tools largely fail to converge with lesion segmentations from a different human expert tracer (systematic comparisons are in the Results below). We sought to address this issue by developing a model for automatic lesion segmentation based on state-of-the-art deep learning techniques. Critically, this process involved evaluating it in a way that is highly rigorous but rarely used. That is, we compared its performance on one set of MRI acquisition sites when it was trained on data from a different set of acquisition sites. Such an evaluation is challenging because models such as ours with many free parameters can easily over-fit the data on which they are trained, leading to poor generalization to new, previously unseen data. We addressed the challenging issue of model over-fitting by performing a rigorous cross-study validation to evaluate accuracy of lesion identification across sites that differ in numerous ways such as scanner model, patient sample, time of scan acquisition after stroke, and use of different expert tracers. Specifically, the process involves training a model on one set of patient MRIs and then testing the ability of those trained parameters to identify lesions in a separate validation (test) set. Cross-study validation gives a better estimate of the model’s true accuracy compared to cross-validation, where train and test samples are simply re-shuffled from the same dataset (Bernau et al., 2014).

Details of the datasets and our model are provided below in the Methods section, followed by experimental results across three different datasets. We show that our system has significantly higher agreement with ground-truth segmentations by human experts compared to the recent CNN-based methods DeepMedic (Kamnitsas et al., 2017), the original UNet (Ronneberger et al., 2015), a residual UNet (Guerrero et al., 2018), and two non-CNN based machine learning methods using either random forests (Pustina et al., 2016) or naive Bayes (Griffis et al., 2016).

## 2 Methods

### 2.1 Imaging data

We obtained whole-brain MRI scans in the form of high-resolution (1 mm^3^) T1-weighted images and FLAIR images with the same in-plane resolution but with 3 mm thick slices. These scans were performed on 25 patients from the Kessler Foundation (KF), a neuro-rehabilitation facility in West Orange, New Jersey. We also obtained 20 such high-resolution scans from the Medical College of Wisconsin (MCW). Data heterogeneity is important for widespread applicability of the model. To that end, we included data from a variety of time points: subacute (1 to 5 weeks post stroke) and chronic ( >  3 months post stroke). Note that despite the inclusion of scans from different post-stroke time points, our current model makes no attempt to track the change in lesions over time. Rather, different time points were included with the aim of enhancing the generalizability of the model. Strokes of both hemorrhagic and ischemic etiology were included. Only cases of left-hemisphere stroke were included. The lesions visualized on the scans were hand-segmented by a trained human expert, as described for the KF scans in (Boukrina et al., 2015) and the MCW scans (Binder, Pillay, Humphries, Gross, Graves, Book, 2016, Pillay, Stengel, Humphries, Book, Binder, 2014).

To move these scans into standard Montreal Neurological Institute (MNI) reference space (Fonov et al., 2011), we used the non-linear warping tool, 3dQwarp, from the AFNI software suite (Cox, 1996). The alignment parameters were calculated first for the T1 images, as their resolution and contrast profile most closely match that of the MNI atlas brain. Those parameters were then applied to align the FLAIR images to MNI space using the AFNI software 3dNwarpApply. As an automatic step in that process, the FLAIR images are resampled to match the dimensions of the MNI atlas reference space. This spatial transformation was also applied to the hand-traced lesion mask. The original segmented lesion was used as an exclusion mask so that the lesioned territory would be excluded from the warping procedure. This prevents non-lesioned brain tissue from being distorted to fill in the lesioned area. This transformation resulted in skull-stripped T1 and FLAIR images, as well as lesion masks, for each patient in MNI space.

Scans and stroke lesion masks were also obtained from the public ATLAS database (Liew et al., 2018). We processed these in the same way as described for the KF and MCW data, with the exception that no FLAIR images were provided in the ATLAS database. Stroke lesions in the MRI scans from this dataset were hand-segmented by different human experts than for the KF and MCW data. These hand-drawn binary masks provide ground-truth for lesion location and extent according to a detailed protocol followed by multiple trained experts (Liew et al., 2018). We selected images according to the following criteria to focus on cases with single lesions in the left hemisphere:


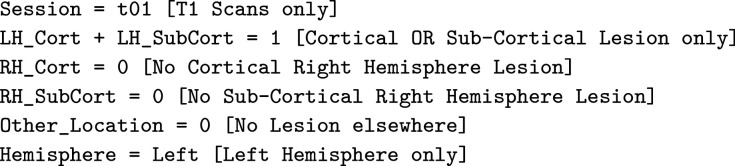


This resulted in 54 images being selected from the ATLAS set. Thus we included a total of 99 images altogether across the three datasets. We divided these into two groups, ATLAS or KF+MCW, for cross-study comparisons. We then combined them to perform a five-fold cross-validation across all 99 images.

### 2.2 Convolutional neural networks

Convolutional neural networks are the current state of the art in machine learning for image recognition (Krizhevsky, Sutskever, Hinton, 2012, LeCun, Bottou, Bengio, Haffner, 1998), including for MRI (Bernal et al., 2019). They are typically composed of alternating layers for convolution and pooling, followed by a final flattened layer. A convolution layer is specified by a filter size and the number of filters in the layer. Briefly, the convolution layer performs a moving dot product against pixels given by a fixed filter of size *k* × *k* (usually 3 × 3 or 5 × 5). The dot product is made non-linear by passing the output to an activation function such as a sigmoid or rectified linear unit (also called relu or hinge) function. Both are differentiable and thus fit into the standard gradient descent framework for optimizing neural networks during training. The output of applying a *k* × *k* convolution against a *p* × *p* image is an image of size (p−k+1)×(p−k+1). In a CNN, the convolution layers just described are typically alternated with pooling layers. The pooling layers serve to reduce dimensionality, making it easier to train the network.

#### 2.2.1 Convolutional U-network

After applying a series of convolutional filters, the final layer dimension is usually much smaller than that of the input images. For the current problem of determining whether a given pixel in the input image is part of a lesion, the output must be of the same dimension as the input. This dimensionality problem was initially solved by taking each pixel in the input image and a localized region around it as input to a convolutional neural network instead of the entire image (Ciresan et al., 2012).

A more powerful recent solution is the Convolutional U-Net (U-Net) (Ronneberger et al., 2015). This has two main features that separate it from traditional CNNs: (a) deconvolution (upsampling) layers to increase image dimensionality, and (b) connections between convolution and deconvolution layers. Another popular U-Net method is the residual U-Net (also known as UResNet (Guerrero et al., 2018)). It has residual connections to prevent the gradient from becoming zero (also called the vanishing gradient problem (Hochreiter, 1998)).

#### 2.2.2 U-Net systems

Since the introduction of the original U-net, several systems have been proposed for analyzing MRI images. DeepMedic is a popular multi-path 3D CNN model that combines high and low resolutions of input images. Previous systems like Lyksborg et al. (2015) consider the three axial, sagittal, and coronal planes in a multi-path ensemble. A potential limitation is that they use a majority vote approach to combine outputs from each path. Multi-path systems can be challenging to train, as can be seen in the work of de Brebisson and Montana (2015). There they train eight networks in parallel to capture various aspects of the input image but report overfitting due to large number of parameters.

Post processing is another important component of U-Net systems to reduce false positives. Post processing methods range from simple ones like connected components and clustering (Havaei, Davy, Warde-Farley, Biard, Courville, Bengio, Pal, Jodoin, Larochelle, 2017, Lai) to using 3D CNNs and conditional random fields (Kamnitsas et al., 2017). The latter methods also end up accounting for dependencies between slices, resulting in a higher accuracy.

### 2.3 Our CNN system

#### 2.3.1 Overview

**Fig. 1:**
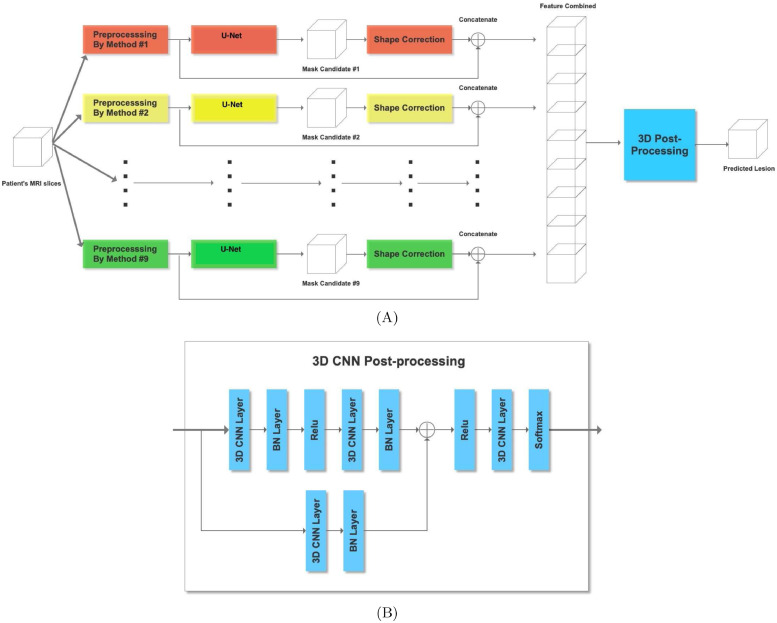
Overview of our entire nine-path system (A) and a zoomed in view of our 3D CNN post processor (B) for combining outputs from each path.

We developed a modified U-network in a multi-path multi-modal system with a 3D convolutional kernel for post-processing shown in Fig. 1. A 3D kernel is like a 2D one except that it has a third dimension that it convolves into as well. For example, in a 2D system kernels are typically 3 × 3 whereas in a 3D kernel it would be 3 × 3 × 3. Details of our system are provided below, highlighting differences in our approach compared to previous ones.

#### 2.3.2 Multiple paths

Our primary motivation for taking a multi-path approach is to optimize the ability of the model to identify brain lesions by capturing image information from all three planes as well as their normalizations. Shown in the overview of our system in Fig. 1(A) are the three different normalizations for each of the three axial, sagittal, and coronal planes. For each plane we normalize (1) in the same plane, (2) across the third plane, and (3) both in the same plane first and then across the third, thus giving nine paths. These choices were motivated by our preliminary results shown in the Supplementary Material. There we see the test accuracy of six paths across nine different samples from the KF dataset. We see that no one path gives the highest accuracy. This has also been shown previously. For example, ensemble methods have been applied where three separate networks are learnt for 2D slices in each of the three axial planes (Lyksborg et al., 2015). This led to improvement over use of a single axial plane. Effects of normalizing on different image views have been recently reviewed (Bernal et al., 2019). Our work here combines the use of both multiple planes and multiple normalizations into a richer model.

#### 2.3.3 Basic U-net

**Fig. 2:**
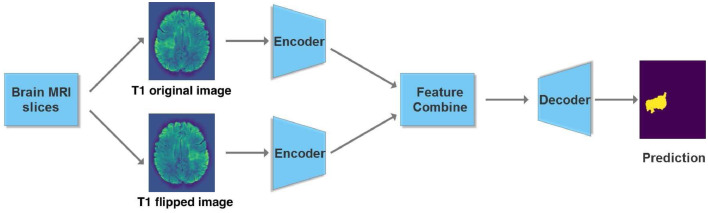
Overview of our dual-path U-network. We have a separate encoder for the original T1 image of the brain scan and one for its flipped version. Alternatively, two different image modalities may also be used instead of two different hemispheres.

*Encoder* First we look at details of our basic U-net shown in Fig. 2 that makes up the system. Since we have dual modality images (T1 and FLAIR), one way to model them in the input is with two channels. An alternative is to have dual paths that allow for specific parameters and thus enhanced model representation for different image modalities. In case the image has a single modality we consider the flipped version as an *augmented* synthetic modality.

**Fig. 3:**
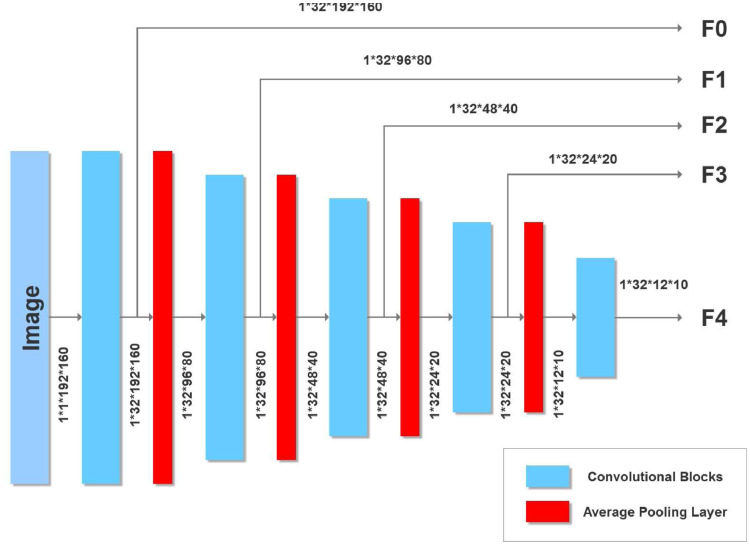
U-Net Encoder with five convolutional blocks. Also shown are image dimensions after each convolution.

**Fig. 4:**
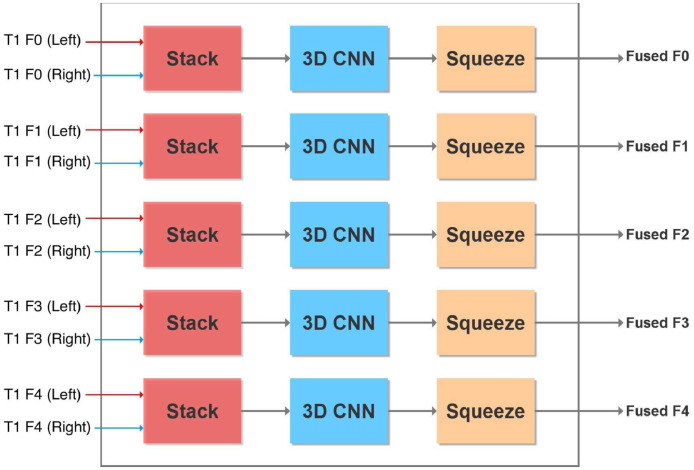
Fuse features from encoding the original and flipped images (or alternatively encoding from two different image formats).

The U-net we use in each path is inspired by the original U-net (Ronneberger et al., 2015) and a more recent one (Tseng et al., 2017) that attains state of the art accuracies on the BRATS brain tumor MRI benchmark (Menze et al., 2014). The encoder portion of our U-net is shown in Fig. 3. After each convolution we perform a 2 × 2 average pooling with stride 2 to halve the image dimension. Features from the encoder are passed to the decoder. However, since there are two encoders (one for the original T1-weighted image and the other for its flipped version), corresponding features are combined using the block shown in Fig. 4. Alternatively, the current network can be used with two different MRI modalities by substituting the T1 image and its flipped version with separate left hemisphere T1-weighted and FLAIR images.

*Feature fusion* From each encoder we obtain a prediction of a lesion (in the respective normalization and plane) that we merge with a 2 × 1 × 1 3D convolutional kernel (Lai, Tseng, Lin, Hsu, Huang, 2017). We take the two feature maps each of dimension 32 × *x* × *y* where 32 is the number of convolutional filters from the encoder layer and *x* × *y* is the input size depending upon the encoder layer (see Figs. 2 and 4). Stacking refers to adding an extra dimension to make the input 32 × 2 × *x* × *y* for the 3D kernel. The 2 × 1 × 1 3D kernel gives an output of 32 × 1 × *x* × *y* which is ”squeezed” to remove the unnecessary dimension to give an output of 32 × *x* × *y* to the decoder.

**Fig. 5:**
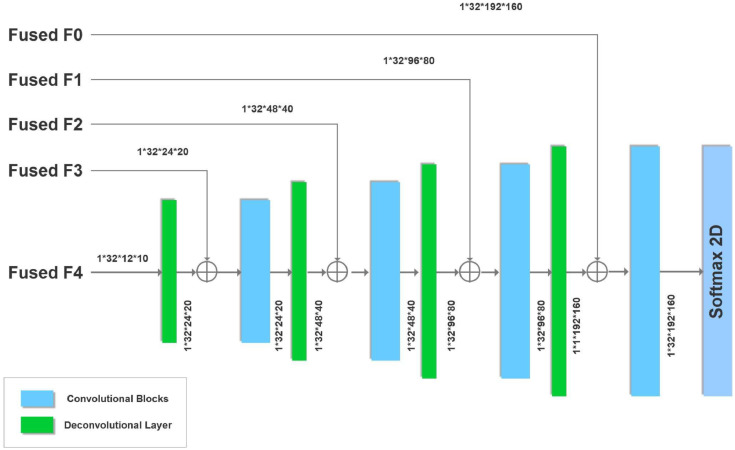
U-Net Decoder with four convolutional and deconvolutional blocks. Also shown are image dimensions after each deconvolution.

*Decoder* The fused features are then given to the decoder, which we add to the output of deconvolutional layers (briefly explained below), a process shown as a ⊕ sign in Fig. 5. The image dimensions are preserved because of the addition. The previous U-net that served as a starting point for our current effort (Tseng et al., 2017) performed element-wise multiplication of fused features with deconvolved ones. However, this is unlikely to be useful for the current system. Our fused features and upsampled features have small values, so their product would even be smaller. This in turn would give a gradient with zero or near-zero values that would affect the training. Thus we prevent this by adding instead of multiplying fused and upsampled feature values.

**Fig. 6:**
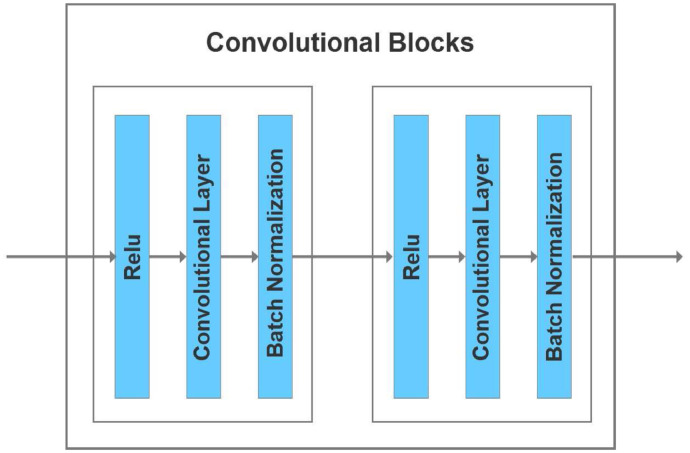
Convolutional blocks used in our encoder and decoder described above.

*Convolutional blocks* Shown in blue in Fig. 6 are the convolutional blocks used in our encoder and decoder. We use 3 × 3 convolutional blocks with a stride of 1 and padding of one extra layer in the input to make the output dimensions match the input. The previous U-net that inspired our design (Tseng et al., 2017) performed Relu activation before adding fused features. Here we perform Relu activation twice. In the context of the decoder, this means Relu activation is performed after adding fused features to upsampled ones. Performing Relu activation after addition rather than before has been shown to be more accurate for image classification (He et al., 2016b).

*Deconvolutional blocks* Deconvolutional blocks (also known as transposed or fractionally strided convolutions) are meant to increase the dimensionality of images (Dumoulin and Visin, 2016). The term transpose arises from the fact that a deconvolution is simply the product of the transpose of the convolution weight matrix with the output when the stride is 1. If the stride is more than one we insert zeros in between the input to obtain the correct transpose result (as explained in (Dumoulin and Visin, 2016)) We use 2 × 2 deconvolutions with a stride of 2 that doubles the image dimensions in both axes.

#### 2.3.4 Post-processing

The output of each of the nine paths in our system is a 2D mask showing the predicted location of the lesion in the same view as the input image, as in Fig. 2. The lesion prediction mask is binarized by rounding to 0 if the values in the mask are below 0.5, otherwise values are rounded up to 1. We stack each predicted lesion with the original input image and combine all slices to form a 2 × 192 × 224 × 192 volume. Since we have nine paths this becomes of size 18 × 192 × 224 × 192. This is passed to our 3D CNN post-processor as described below.

In the post-processor shown in Fig. 1(B), we have a main path containing 36 3D 3 × 3 × 3 kernels each with 18 channels, or equivalently 36 3D kernels each of size 18 × 3 × 3 × 3. Following that, the second 3D CNN in the main path has 9 3D 3 × 3 × 3 kernels each with 36 channels, and two final 3D CNNs each of dimensions 3 × 3 × 3 with 9 channels.

#### 2.3.5 Loss function

The final output from the post-processor has two channels each of dimensions 192 × 224 × 192. The target lesion has the same dimensions but just one channel. The first channel in our output predicts the lesion and the second one predicts the complement of it. We convert the outputs of each channel into probabilities with softmax (Alpaydin, 2004) and combined them into a modified Dice loss function (Milletari, Navab, Ahmadi, 2016, Wong, Moradi, Tang, Syeda-Mahmood, 2018). For a single channel output the Dice loss is defined to be 1−D where

$$D\left(p\right)=\frac{2\sum_{i}p_{i}r_{i}}{\sum_{i}p_{i}^{2}+\sum_{i}r_{i}^{2}}$$

*p_i_* are the predicted softmax outputs of the channel, and *r_i_* is 1 if the voxel has a lesion and 0 otherwise. If we are predicting the complement of the lesion then the values of *r_i_* are flipped from 0 to 1 and 1 to 0. With our two channel output *p* and *q* our loss becomes 2−(D(p)+D(q)) where the latter *D*(*q*) is for the complement.

### 2.4 Comparison of CNN methods

We compared our CNN to three recently published CNNs shown below. Our system was implemented using Pytorch (Paszke et al., 2017), the source code for which is available on our GitHub site https://github.com/xyzacademic/multipathbmp. In each of our experiments we train our model, UNet, and UResNet with stochastic gradient descent and Nesterov momentum (Ruder, 2016) of 0.9 and weight decay of 0.0001. We use a batch size of 32, starting from an initial learning rate of 0.01 with a 3% weight decay after each epoch for a total of 50 epochs. In DeepMedic we use the default settings of learning rate of 0.001, the RMSProp optimizer (Ruder, 2016) with a weight decay of 0.0001, batch size of 10, and a total of 20 epochs.

• DeepMedic (Kamnitsas et al., 2017): This is a popular dual-path 3D convolutional neural network with a conditional random field to account for temporal order of slices. DeepMedic contains a path for low- and a separate path for high-resolution of images. Its success was demonstrated by winning the ISLES 2015 competition to identify brain injuries, tumors, and stroke lesions. The code for implementing DeepMedic is freely available on GitHub, https://github.com/Kamnitsask/deepmedic.
• UResNet (Guerrero et al., 2018): This is a convolutional neural network with residual connections (He et al., 2016a). The code for implementing UResNet is also freely available on GitHub, https://github.com/DeepLearnPhysics/pytorch-uresnet.
• UNet (Ronneberger et al., 2015): The was the original convolutional U-network proposed for biomedical image processing. Its code is also available on GitHub,
  https://github.com/thonycc/PFE/tree/af9e804f71684b73cf7f3b25557edcf6a1b307b3.

Two other non-CNN-based machine learning packages were also included because they have been made freely available to the brain imaging community and have been developed for ease of use. Both take a patch-based approach to automating lesion segmentation. That is, these methods convert the input image into multiple patches that are used to train the model. They are LINDA (Pustina et al., 2016), based on a random forests algorithm, and a second method based on Gaussian naive Bayes (Griffis et al., 2016).

### 2.5 Data analysis

#### 2.5.1 Measure of accuracy: Dice coefficient

The Dice coefficient is typically used to measure the accuracy of predicted lesions in MRI images (Zijdenbos et al., 1994). The output of our system and that of other methods is a binary mask of the dimensions as the input image, but with a 1 for each voxel calculated to contain a lesion, and a 0 otherwise. Comparison of the human expert-segmented lesion mask with that from automated methods is quantified with the Dice coefficient. Starting with the human binary mask as ground truth, each predicted voxel is determined to be either a true positive (TP, also 1 in true mask), false positive (FP, predicted as 1 but 0 in the true mask), or false negative (FN, predicted as 0 but 1 in the true mask). The Dice coefficient is formally defined as

(1) $$DICE=\frac{2TP}{2TP+FP+FN}$$

#### 2.5.2 Measure of statistical significance: Wilcoxon rank sum test

The Wilcoxon rank sum test (Wilcoxon, 1945) (also known as the Mann-Whitney *U* test) can be used to determine whether the difference between two sets of measurements is significant. It is a non-parametric test for whether two sets of observations are likely to be from different distributions, without assuming a particular shape for those distributions. Formally, it tests for the null hypothesis that a randomly selected point from a sample is equally likely to be lower or higher than a randomly selected one from a second sample.

## 3 Results

In the results presented below, we take the rare and rigorous step of performing cross-study validations across independent datasets (Bernau et al., 2014). We also examine results from cross-validation in the combined dataset from the three different sources (KF, MCW, and ATLAS).

### 3.1 Cross-study validation results

To create relatively balanced sets in terms of number of T1 scans, we combine the KF and MCW datasets into one. This yielded 45 samples in KF+MCW and 54 in ATLAS. We first train all convolutional neural networks (CNNs) on the KF+MCW data and test their ability to predict lesion locations in the ATLAS set. We then repeat the same procedure but with the train and test datasets reversed. Since LINDA and GNB come pre-trained and were intended for out-of-the-box use rather than re-training, we ran them as-is. Both programs have skull-removal built into their pipelines. Because the ATLAS images were the largest dataset with the skull still intact, we restricted our test of the LINDA and GNB methods to the ATLAS dataset.

#### 3.1.1 Train on KF+MCW, predict on ATLAS

**Fig. 7:**
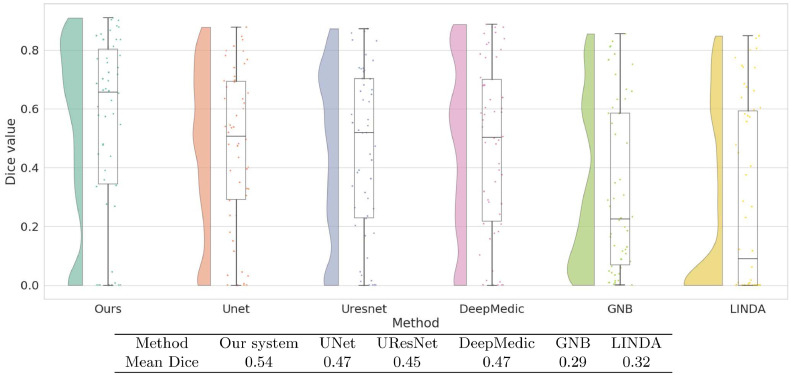
Raincloud plots of Dice coefficient values of all models trained on KF+MCW and tested on ATLAS. For each method we show the distribution of Dice coefficients across all test images as well as the five summary values: median (middle horizontal line), third quartile (upper horizontal line), first quartile (lower horizontal line), min (lowermost bar), and max (uppermost bar). All models except for LINDA and GNB are trained on KF+MCW. The Table below the graph contains the mean Dice coefficients of all models on the ATLAS test data.

Fig. 7 shows the Dice coefficient values on the ATLAS test dataset with training performed on KF and MCW images. Results show that the current system, with a median Dice value of 0.66, yielded the best performance. This was not just due to a few high values, as its Dice values generally clustered toward the higher end. The Dice values of UNet, UResNet, and DeepMedic Dice have a more even distribution than our system and lower median values. Both LINDA and GNB have Dice values clustered toward the lower end. Fig. 7 also shows that our system has the highest mean Dice value of 0.54. This value is reliably higher than all other methods under the Wilcoxon rank test (p  <  0.001). All the convolutional networks achieve better median values than LINDA and GNB.

#### 3.1.2 Train on ATLAS, predict on KF+MCW

**Fig. 8:**
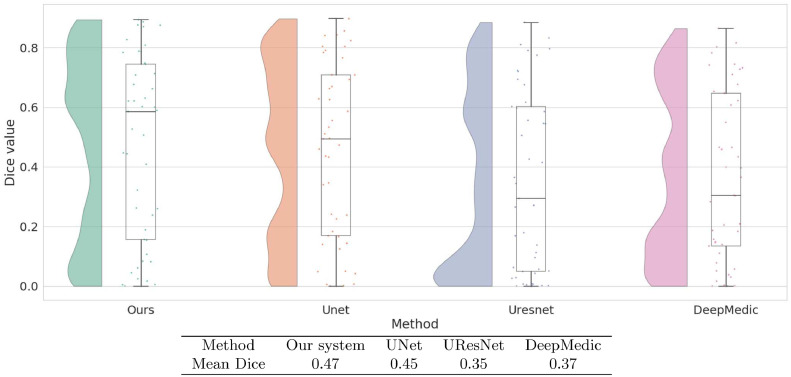
Raincloud plots of Dice coefficient values for all models trained on ATLAS and tested on KF+MCW. Also shown in the table are mean Dice coefficients of each method, as tested on the KF+MCW set.

Fig. 8 shows results from the other direction of the cross-study analysis: training on ATLAS and testing on KF+MCW. In this case, although our system has the highest median, its distribution of Dice values is no longer clustered toward the high end as it was previously. The mean Dice value of our system is marginally above that of UNet alone and not statistically distinguishable from it. Compared to UResNet and DeepMedic, however, our method performs better, as shown from its reliably higher Dice values (p  <  0.001).

### 3.2 Cross-validation results on all datasets ATLAS, KF, and MCW combined

**Fig. 9:**
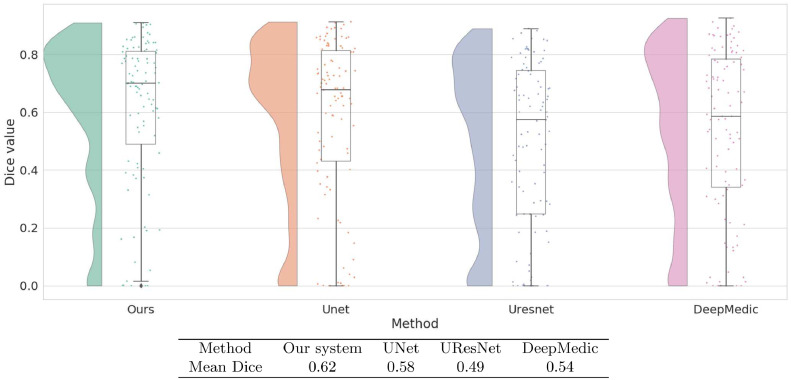
Raincloud plot of Dice coefficient values obtained by five-fold cross validation on all our data combined: ATLAS+Kessler+MCW. In the Table are the mean Dice coefficients given by cross-validation.

To take full advantage or our relatively large dataset, we combined images from all three sources to produce an overall dataset of 99 samples. We then performed a five-fold cross-validation on this combined dataset to evaluate the accuracy of each method. Fig. 9 shows that our system again has the highest median Dice value. Our system also has the highest mean Dice value at 0.62, performing reliably better than the next best system, UNet, at 0.58. Indeed, our system performed better (p  <  0.001) than all three of the other CNN-based systems.

**Fig. 10:**
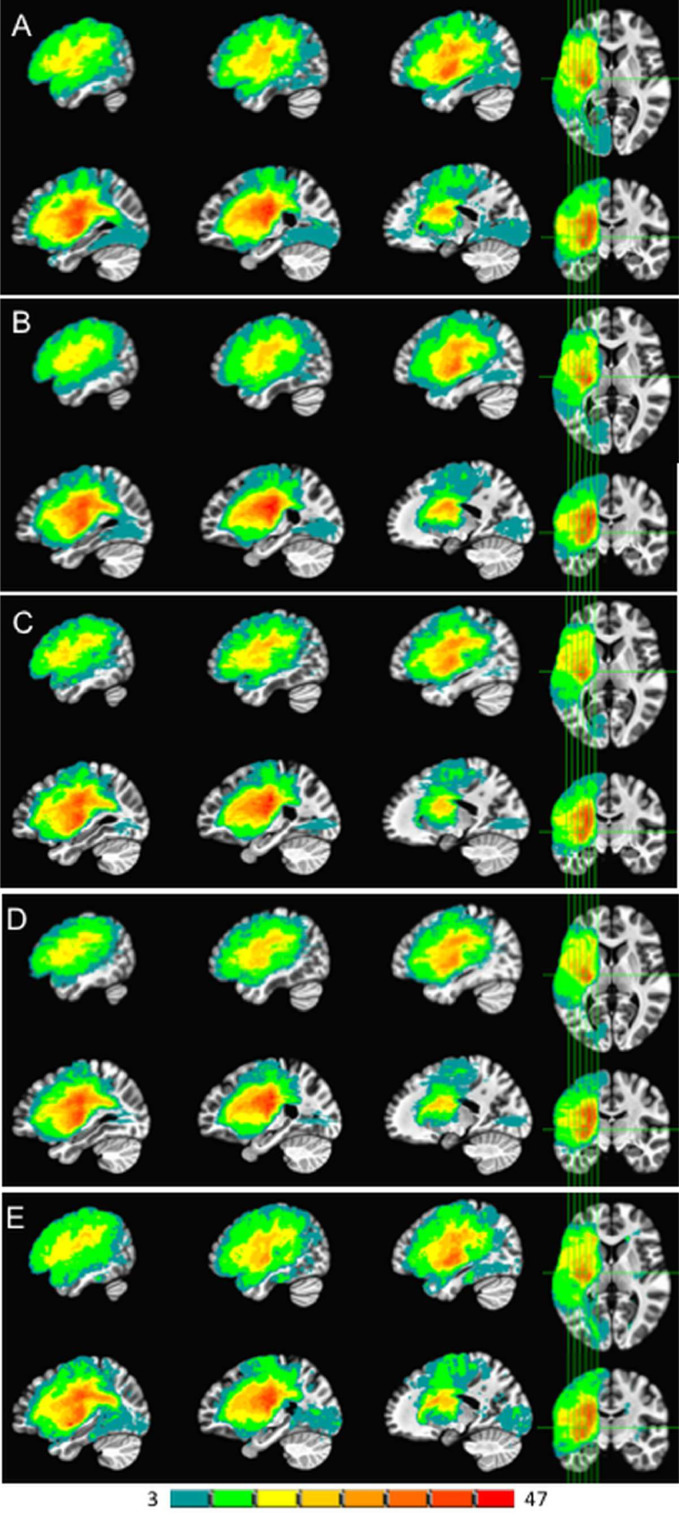
Lesion overlap map results from 5-fold cross-validation on the entire 99 scan dataset. The leftmost side of the color scale in teal shows locations with 3 spatially overlapping lesions, while the rightmost side in red shows a maximum of 47 overlapping lesions. Hand-segmented lesions are in panel A. Our 2.5D CNN model is in panel B, UNet in C, URestNet in D, and DeepMedic in E. (For interpretation of the references to colour in this figure legend, the reader is referred to the web version of this article.)

In addition to reporting this advantageous numeric performance of our system, an overall illustration of how the lesion masks produced by the current model compared to those from the other CNN-based models is in Fig. 10. The expert-traced lesions (A) are shown alongside those produced by our system (B) and the other models (C-E).

One point to note is that while our system performed significantly better in terms of overlap with human expert tracings as measured by the Dice coefficient, visually all the automatic methods appear grossly similar to the human expert segmentations.

### 3.3 Distribution of dice coefficients across lesion size

**Fig. 11:**
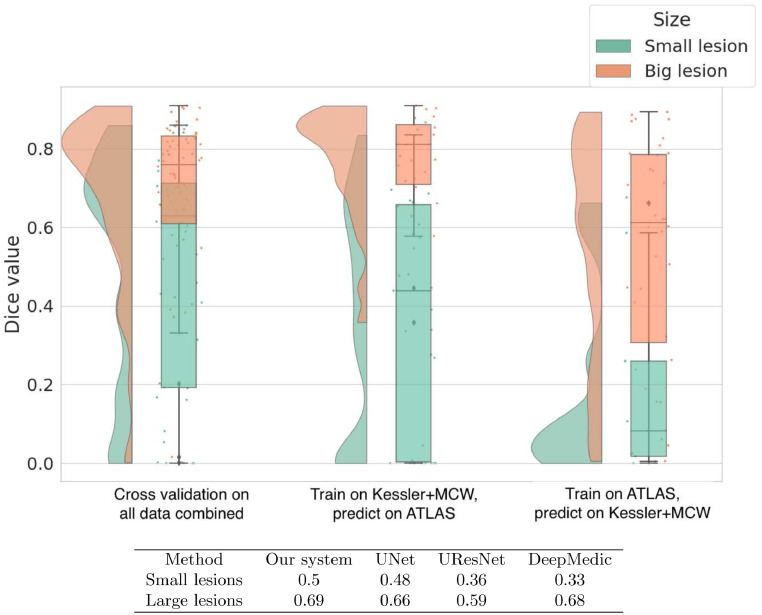
Raincloud plot showing the distribution and five summary statistics of Dice coefficients in three different scenarios. The left panel shows Dice values given by cross-validation on all the data combined. The middle panel shows a cross-study scenario where the current model is trained on KF+MCW and tested on ATLAS. The right panel shows results from training on ATLAS and testing on KF+MCW. In the Table below the plots we show the mean Dice values of our system and the other CNNs on small and large lesions separately in cross-validation on all data combined.

Lesions with *x* × *y* × *z* dimensions less than 20 × 20 × 25 mm were classified as small, and any lesions with dimensions greater than those were considered large. In Fig. 11 we show a raincloud plot of Dice values obtained by our system in the cross-validation and cross-study settings.

Smaller lesions are generally harder to identify than larger ones Griffis et al. (2016); Ito et al. (2019); Pustina et al. (2016). To compare performance between lesion sizes, we split the lesions into small and large categories based on the distribution of lesion sizes in the overall set.

**Fig. 12:**
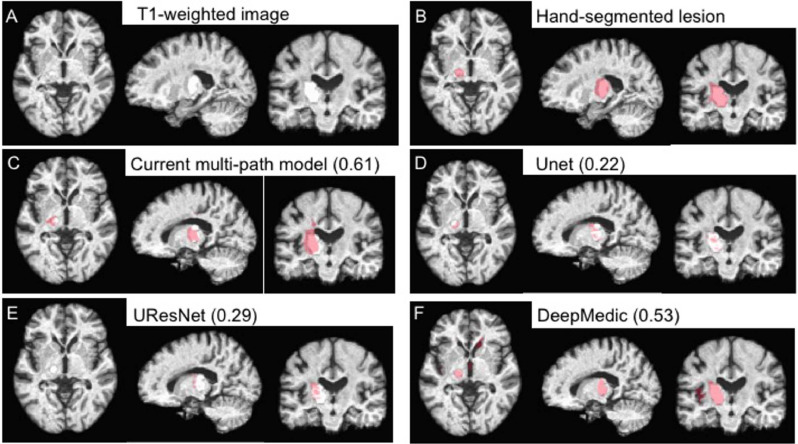
Example of a relatively large (10,739 mm^3^) lesion (A) along with its hand-segmented mask (B). The remaining panels show the lesion masks derived from the 5-fold cross-validation with all 99 scans for our 2.5D model (C) and the other CNN-based approaches (D-F). The label for each model is followed by the corresponding Dice value for the lesion mask it produced in parentheses. Lesion masks overlaid in red are rendered semi-transparent to visualize the overlap between the lesion and the mask. (For interpretation of the references to colour in this figure legend, the reader is referred to the web version of this article.)

In all three cases, our method does very well on large lesions. In fact, when we train on KF+MCW and predict on ATLAS, the median Dice is above 0.8 for large lesions. In the cross-validation on all data combined, our model is significantly better than all methods except for DeepMedic, with p-values below 0.05. An example of a larger lesion is shown in Fig. 12. The output lesion masks in red show our method and the other three to be qualitatively similar. An apparent exception is DeepMedic, which misidentifies tissue in the right-hemisphere as being lesioned. This mis-identification would seem to be an exception, however, given the similar numeric performance between our method and DeepMedic.

**Fig. 13:**
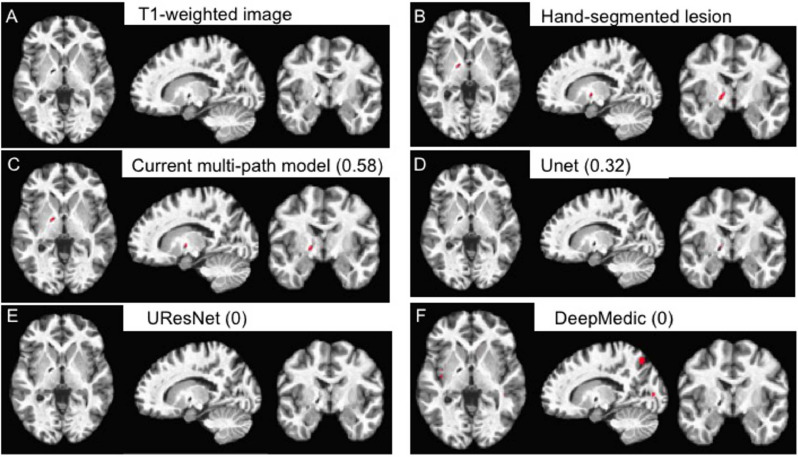
Example of a relatively small (85 mm^3^) lesion (A) along with its hand-segmented mask (B). The remaining panels show the lesion masks derived from the 5-fold cross-validation with all 99 scans for our 2.5D model (C) and the other CNN-based approaches (D-F). The label for each model is followed by the corresponding Dice value for the lesion mask it produced in parentheses. Lesion masks are overlaid in red. Note that the lesion masks derived from the DeepMedic model (F) are false positives rather than actual lesions. (For interpretation of the references to colour in this figure legend, the reader is referred to the web version of this article.)

Smaller lesions, on the other hand, are associated with lower median Dice values overall, as generally expected. DeepMedic has particular difficulty with smaller lesions, whereas our system shows significantly greater accuracy than DeepMedic and UResNet. Interestingly, the distribution of Dice values for small lesions clusters towards the high end in the cross-validation setup with the most training data (all three datasets combined). This suggests that still more data would enable the model to achieve better accuracy at identifying small lesions. An example of a smaller lesion classification for the combined data cross-validation scenario is shown in Fig. 13. This figure shows how the similarity of the overall contours of the model-based lesion masks (C-F) match up with the hand-segmented lesion mask (B). It also illustrates the face validity of the Dice coefficient, where higher Dice values also qualitatively correspond better to the hand-segmented lesion mask.

CNNs are a type of neural network, and what neural networks learn depends on what information is in the training data Plaut et al. (1996). In the cross-study scenario where we train on KF+MCW and test on ATLAS, the distribution of Dice values for smaller lesions is spread somewhat uniformly. However, when the network is trained on the ATLAS data and tested on the KF+MCW set, performance is worse. Thus the general rule that the information in the training dataset largely determines what the model can learn is also shown here for detecting small lesions.

### 3.4 Consolidating multi-path outputs

**Fig. 14:**
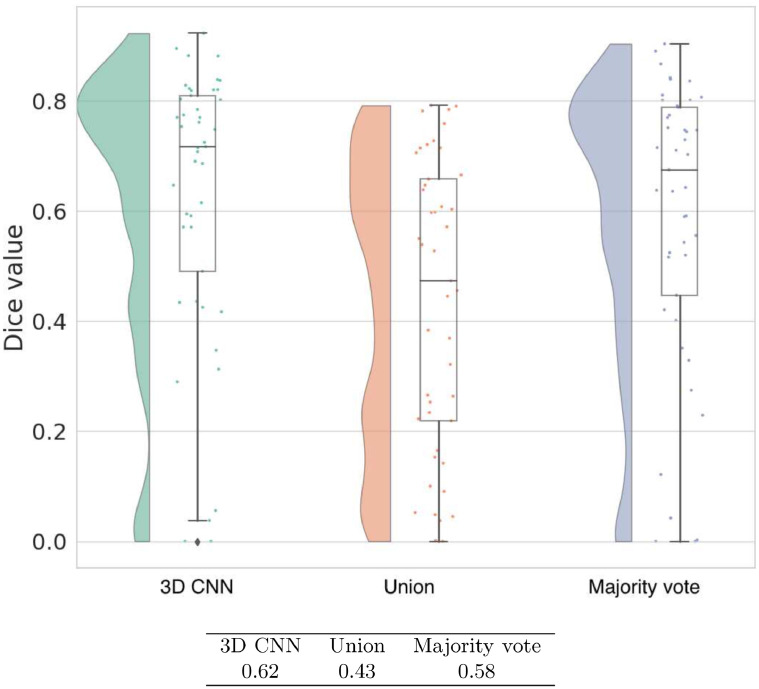
Raincloud plot of Dice coefficient values of three different post-processing approaches in our system as given by five-fold cross validation on KF+MCW images combined. Mean Dice values for each approach are presented in the accompanying table.

Previous multi-path approaches use a majority vote to combine outputs from different paths (Lyksborg et al., 2015). We compare our 3D CNN for combining multi-path outputs to using the majority vote and a simple union. In the union method, if at least one pixel has a 1 across the paths then the aggregated output also has a 1 in that pixel. Fig. 14 shows that the union clearly performs more poorly than majority vote and our 3D CNN. Between the two better performing methods, the 3D CNN is reliably better than majority vote by a 4% margin with a p-value of 0.004. Also compared to post-processing with majority vote, the Dice values of the 3D CNN are concentrated more towards the high end.

### 3.5 Multi-modal T1 vs. T1+FLAIR

Our basic U-Net model is multimodal (specifically, bimodal) in that it allows for different image formats. Since the current project is focused exclusively on left hemisphere lesions, we present the model with T1 and FLAIR image formats of the lesioned left hemisphere. Below in Table 1 we show the cross-validation accuracy of our model on the KF and MCW images. When presented together, there is no significant difference between the two. However, if we look at just KF images that contain smaller lesions (and more recent, in the less than 5-week post-stroke range), then adding FLAIR confers a significant advantage. In the case of MCW images only that have lesions exclusively in the chronic epoch (at least 6 months post-stroke), the T1 images alone actually result in better performance than when the corresponding FLAIR images are added. This pattern corresponds with the standard clinical observation that FLAIR scans are useful for more recent stroke lesions but less so for those in the chronic phase (Ricci et al., 1999). Such correspondence lends additional face validity to our model.

### 3.6 Training and inference runtimes

The time for our model, UNet, and UResNet to take an image and output its predicted lesion (also known as inference time) is less than a second. For DeepMedic the inference time is longer (but still in the order of seconds) because it divides the image into patches and evaluates each patch in the model. The training times of all models however are much longer, on the order of hours. This is typical for deep learning models. In Table 2 we show training times for our model on the ATLAS dataset and inference time for a single patient. Each of our nine paths was trained in parallel to reduce runtime.

**Table 2:** Total training runtime on ATLAS and inference time on a single patient for all models in our study.

	Our system	UNet	UResNet	DeepMedic
Total train time on ATLAS	35 mins per path (total is 315 mins)	75 mins	45 mins	12 h
Time for inference on a single patient	0.5 s	0.9 s	0.7 s	15 s

## 4 Discussion

Here we have created, trained, and tested a new multi-path 2.5D convolutional neural network. The fractional designation on the dimension comes from its use of nine different 2D paths, followed by concatenation of the learned features across the paths, which are then passed to a 3D CNN for post-processing. This 2.5D design combines flexible and efficient 2D paths that process the data in different canonical orientations and normalizations with a 3D CNN that combines the 2D features in a way that informs the final 3D image output. Comparison of our system to previous efforts shows that CNN-based systems outperform more traditional machine-learning approaches based on random forests or Gaussian naive Bayes algorithms. Compared to other CNN systems, our system shows reliably superior performance in its ability to automatically segment stroke lesions from healthy tissue in the left hemisphere.

To facilitate comparisons across multiple different CNN-based models and machine learning methods, we used identical input images for all models. Specifically, this facilitated comparison across a combination of basic machine learning methods like Naive Bayes (Griffis et al., 2016) and random forests (Pustina et al., 2016), and more sophisticated convolutional neural networks such as the U-Net (Ronneberger et al., 2015). Compared to the more general purpose classifiers of naive Bayes and random forests, the CNN-based models offer a greater number of parameters and techniques designed for image processing such as convolutions (LeCun et al., 1998). As expected, Dice accuracies were generally greater for the models with larger numbers of parameters.

The CNNs considered here include a 3D model, DeepMedic (Kamnitsas et al., 2017), and two 2D models, which are the original U-Net (Ronneberger et al., 2015) and the U-ResNet (Guerrero et al., 2018). There are pros and cons to each. While 3D gives greater flexibility in modeling the data by providing more parameters, it also requires more data to avoid overfitting, as reflected in the relatively poor cross-study performance of DeepMedic. A 2D model on the other hand may not be sufficiently sensitive to the spatial information present in the 3D input images. Our current 2.5D model strikes a balance by using a combination of 2D and 3D convolutions. Here we achieved higher Dice accuracy than comparison models by using 2D components for different view planes of the 3D scan and a 3D kernel to merge the views into a voxel segmentation for the output.

As methods for automated segmentation of brain lesions continue to develop, a question arises. How good is good enough? An intuitive answer comes from human expert raters. As mentioned in the Introduction, human expert raters have been shown to produce lesion segmentations with overlapping volumes between raters in the 67% to 78% range (Fiez, Damasio, Grabowski, 2000, Liew, Anglin, Banks, Sondag, Ito, Kim, Chan, Ito, Jung, Khoshab, et al., 2018, Neumann, Jonsdottir, Mouridsen, Hjort, Gyldensted, Bizzi, Fiehler, Gasparotti, Gillard, Hermier, et al., 2009), though 73% may be a more realistic upper value given the highly expert raters and limited scope of the data used by (Neumann et al., 2009) to obtain the 78% value. The Dice coefficient used here is a formal measure of degree of spatial overlap that ranges between 0 and 1. Therefore a Dice coefficient in the 0.67 range can be considered to be at the edge of the human expert gold standard. When combining the datasets and performing iterative training and testing using standard 5-fold cross-validation, the lesion traces from our model overlap with human experts with a mean Dice coefficient of 0.62. While the 0.67 to 0.73 human benchmark range should be interpreted with caution because those numbers are based on data that are not identical to the data considered here, the accuracy of our system relative to previous efforts does suggest that deep learning-based CNN methods are beginning to approach human expert level accuracy for stroke lesion segmentation.

Aside from comparison to human expert benchmarking, we can roughly compare our results to recent studies that also use the ATLAS database for evaluation. Qi et al. (2019) report a 5-fold cross-validation Dice accuracy of 0.49 with their depthwise separable convolutional network on the entire ATLAS dataset of 229 images. Similarly Zhou et al. (2019) report a Dice value of 0.53 on a single 5-fold split with their dimension fusion convolutional network also on 229 ATLAS samples. In comparison, we obtain a 5-fold cross-validate Dice accuracy of 0.63 on the 54 ATLAS samples that we use. While this informal comparison is not a statistical result, it is encouraging for our model to see a high Dice value surpassing other recent results.

### 4.1 Future directions

An alternative to our system is to have a multi-modal 3D U-Net instead of the current 2D ones. It may appear that a 3D model would be a better option but there are advantages to a 2.5D model stemming mainly from its simplicity. As noted above, a fully 3D model may overfit the data since it has more parameters and require significantly more computational resources in training. Here we show that our model can generalize across different data sources. This may primarily be due to the 2.5D nature since the 3D component in our model is simply used to combine output from different modalities. A preliminary fully 3D model that we have implemented separately performs far more poorly across different datasets. Thus while promising, a fully 3D CNN model may be difficult to successfully work in practice. Training a 3D CNN involves adjusting many more parameters than for a 2D CNN, and would therefore require more data to train.

A second future direction is to extend our current left hemisphere-focused system to include lesions to the right hemisphere. This extension should be relatively straightforward, as nothing is preventing our current system from being trained and tested on images with lesions to either hemisphere.

The majority of brain lesion-deficit studies involving stroke survivors are performed with participants in the chronic epoch, when most post-stroke brain changes are thought to have stabilized (Damasio, Tranel, Grabowski, Adolphs, Damasio, 2004, Karnath, Rennig, 2017). It is to facilitate such studies that we have largely focused on automatically segmenting stroke lesions from T1-weighted MRI scans. A natural extension of this work would be to track changes in the lesion over time. Maximal changes would presumably occur relative to the acute stage (often defined as  < 48 hours post stroke), for which diffusion-weighted imaging (DWI) would be useful (Karnath, Rennig, 2017, Ochfeld, Newhart, Molitoris, Leigh, Cloutman, Davis, Crinion, Hillis, 2010). Careful attention would presumably need to be paid to acquiring data such as DWI to facilitate the tracking of changes in stroke lesion volume over time. Indeed, another source of brain pathology that changes over time and to which CNN models have been applied with some success is tumor. Studies such as that of (Duong et al., 2019) suggest that training on numerous types of brain pathologies imaged with FLAIR, including tumor, can yield Dice coefficients between human and model segmentations on the order of 0.79. This apparently high level of overlap points to the potential of a multi-pathology approach.

Other progress can likely be made even when sticking to standard T1-weighted MRI. For example, we have concrete plans to explore the use of synthetic multi-modalities by adding images generated from a generative adversarial network (Goodfellow et al., 2014) or similar images from a reference database. This would give our model additional views and information about the input images that may potentially increase its accuracy. Another future avenue is to output confidence values in the predictions as a step towards a fully automated system. A naive approach of simply averaging the probabilities in the predicted lesion as a confidence value does not work because the prediction image contains values close to 0 or 1. Thus the confidence would simply be the size of the predicted lesion. This requires additional work that we plan to explore going forward.

Because the initial application case we envisioned for this work was research, we focused on research-quality scans. These isotropic T1 scans are typically of higher resolution of 1 mm^3^ compared to non-isotropic and 2D scans that have lower out-of-plane resolution, often on the order of 5*mm* thickness between slices. Automatically segmenting lesions from such lower-resolution images (such as many clinical images) would be a challenge. One way to address this is with generative approaches that can increase image resolution. For example Ledig et al. (2017) introduce a generative model to increase resolution of images that could potentially work for medical imaging as well. In separate future work we plan to investigate non-isotropic scans, such as using generative adversarial models (Goodfellow et al., 2014) to convert non-isotropic to isotropic ones.

Another challenge is image noise. Our studies have so far considered only relatively clean images with minimal noise. Some images in our data do have more noise relative to others. Thus our approach may already be robust to some degree of image noise. However, a systematic exploration of this issue is beyond the scope of the current study and would be the subject of important future work.

Small lesions are also a challenge. They may be hard even for a trained practitioner to detect, so it was not surprising that they were difficult to detect for our model and the others. We plan to address this in future work by adding more samples with small lesions as additional datapoints. We will explore generative models as a source of such additional images. The challenge is not just to be able to generate MRI images but to simultaneously generate their correct lesion map as well so the images can be used to help train the model.

Finally, in terms of research and clinical applications, a direct test of the usefulness of the automatic stroke lesion segmentation model would involve applying it in studies of lesion-deficit relationships. As noted in the Introduction, hand-segmentation of brain lesions by human experts is the current gold standard, although some studies have begun relying entirely on computer-generated lesion segmentations (Tyler, Marslen-Wilson, Stamatakis, 2005, Woollams, Halai, Ralph, 2018). Human inter-rater reliability presumably also represents the upper limit for inter-rater reliability between model-based and human expert-generated lesion segmentations. To our knowledge, however, it remains unknown whether the error profile for humans is comparable to that of deep-learning based models such as ours. This raises the question of whether such error would lead to systematic differences in results from lesion-deficit analyses based on either human or machine-segmented lesions. Such comparisons are a concrete future direction of this work.

### 4.2 Conclusion

We have presented a multi-path, multi-modal convolutional neural network system for identifying lesions in brain MRI images. Our method is fully automatic. Given an input MRI image it outputs the lesion without any human intervention. We show that our model achieves significantly higher accuracies than several previous machine learning methods (including other convolutional neural networks) on a cohort of three different datasets. Our cross-study result also rigorously demonstrates that our model generalizes across different datasets. In terms of usability, our model inference times are in seconds, which make it fast to use in practice.

While the data with which our model is trained and tested includes exclusively left-hemisphere lesions, our model can be trained and tested on lesions present anywhere in the brain. In cross-study and cross-validation tests, our model shows superior performance compared to existing CNN and non-CNN based machine learning methods for lesion identification. Our method extends previous efforts showing relatively high segmentation accuracy for large lesions. Given sufficient data, it markedly improves on previous efforts by being able to segment smaller lesions as well. We provide freely available open source code to train and test our model.

This advance in performance is critically significant, as it brings the field closer to removing the bottleneck of having human experts spend numerous hours hand-segmenting stroke lesions on MRI brain scans. Once automated methods are sufficiently accurate and widely available, they will free up researchers to focus their time on other critical aspects of neuropsychological data acquisition and analysis. The hope is this re-allocation of expert resources will help advance the pace at which we can further our understanding of the critical neural bases of cognition and behavior.

## CRediT authorship contribution statement

**Yunzhe Xue:** . **Fadi G. Farhat:** . **Olga Boukrina:** . **A.M. Barrett:** . **Jeffrey R. Binder:** . **Usman W. Roshan:** Writing - original draft. **William W. Graves:** Writing - original draft.

## Appendix A Supplementary materials

Supplementary Data S1 Supplementary Raw Research Data. This is open data under the CC BY license http://creativecommons.org/licenses/by/4.0/ Supplementary Data S1

## References

[bib0001] Akkus Z., Galimzianova A., Hoogi A., Rubin D.L., Erickson B.J. (2017). Deep learning for brain MRI segmentation: state of the art and future directions. J. Dig. Imaging.

[bib0002] Alpaydin E. (2004). Machine Learning.

[bib0003] Aslan M.S., Shalaby A., Abdelmunim H., Farag A.A. (2013). Probabilistic shape-based segmentation method using level sets. IET Comput. Vis..

[bib0004] Aslan M.S., Shalaby A., Farag A.A. (2013). Clinically desired segmentation method for vertebral bodies. Proceedings of the IEEE 10th International Symposium on Biomedical Imaging.

[bib0005] Bernal J., Kushibar K., Asfaw D.S., Valverde S., Oliver A., Martí R., Lladó X. (2019). Deep con- volutional neural networks for brain image analysis on magnetic resonance imaging: a review. Art. intel. med..

[bib0006] Bernau C., Riester M., Boulesteix A.-L., Parmigiani G., Huttenhower C., Waldron L., Trippa L. (2014). Cross-study validation for the assessment of prediction algorithms. Bioinformatics.

[bib0007] Binder J.R., Pillay S.B., Humphries C.J., Gross W.L., Graves W.W., Book D.S. (2016). Surface errors without semantic impairment in acquired dyslexia: a voxel-based lesion–symptom mapping study. Brain.

[bib0008] Boukrina O., Barrett A., Alexander E.J., Yao B., Graves W.W. (2015). Neurally dissociable cognitive components of reading deficits in subacute stroke. Front. Hum. Neurosci..

[bib0009] de Brebisson A., Montana G. (2015). Deep neural networks for anatomical brain segmentation. Proceedings of the IEEE Conference on Computer Vision and Pattern Recognition Workshops.

[bib0010] Ciresan D., Giusti A., Gambardella L.M., Schmidhuber J. (2012). Deep neural networks segment neuronal membranes in electron microscopy images. Proceedings of the Advances in Neural Information Processing Systems.

[bib0011] Cox R.W. (1996). AFNI: software for analysis and visualization of functional magnetic resonance neuroimages. Comput. Biomed. Res..

[bib0012] Damasio H., Tranel D., Grabowski T., Adolphs R., Damasio A. (2004). Neural systems behind word and concept retrieval. Cognition.

[bib0013] Dumoulin, V., Visin, F., A guide to convolution arithmetic for deep learning, arXiv preprint arXiv:1603.07285, 2016.

[bib0014] Duong M., Rudie J., Wang J., Xie L., Mohan S., Gee J., Rauschecker A. (2019). Convolutional neural network for automated flair lesion segmentation on clinical brain mr imaging. Am. J. Neuroradiol..

[bib0015] Farag A.A., El-Baz A.S., Gimel’farb G. (2006). Precise segmentation of multimodal images. IEEE Trans. Image Process..

[bib0016] Fiez J.A., Damasio H., Grabowski T.J. (2000). Lesion segmentation and manual warping to a reference brain: Intra- and interobserver reliability. Hum. Brain Mapping.

[bib0017] Fonov V., Evans A.C., Botteron K., Almli C.R., McKinstry R.C., Collins D.L., Group B.D.C. (2011). Unbiased average age-appropriate atlases for pediatric studies. Neuroimage.

[bib0018] Freund Y., Schapire R.E. (1997). A decision-theoretic generalization of on-line learning and an application to boosting. J. Comput. Syst. Sci..

[bib0019] Goodfellow I., Pouget-Abadie J., Mirza M., Xu B., Warde-Farley D., Ozair S., Courville A., Bengio Y. (2014). Generative adversarial nets. Proceedings of the Advances in Neural Information Processing Systems.

[bib0020] Griffis J.C., Allendorfer J.B., Szaflarski J.P. (2016). Voxel-based gaussian Naïve Bayes classification of ischemic stroke lesions in individual t1-weighted MRI scans. J. Neurosci. Methods.

[bib0021] Guerrero R., Qin C., Oktay O., Bowles C., Chen L., Joules R., Wolz R., Valdés-Hernández M., Dickie D., Wardlaw J. (2018). White matter hyperintensity and stroke lesion segmentation and differentiation using convolutional neural networks. NeuroImage: Clin..

[bib0022] Havaei M., Davy A., Warde-Farley D., Biard A., Courville A., Bengio Y., Pal C., Jodoin P.-M., Larochelle H. (2017). Brain tumor segmentation with deep neural networks. Med. Image Anal..

[bib0023] He K., Zhang X., Ren S., Sun J. (2016). Deep residual learning for image recognition. Proceedings of the IEEE Conference on Computer Vision and Pattern Recognition.

[bib0024] He K., Zhang X., Ren S., Sun J. (2016). Identity mappings in deep residual networks. Proceedings of the European Conference on Computer Vision.

[bib0025] Hochreiter S. (1998). The vanishing gradient problem during learning recurrent neural nets and problem solutions. Int. J. Uncertain. Fuzziness Knowl. Based Syst..

[bib0026] Ito K.L., Kim H., Liew S.-L. (2019). A comparison of automated lesion segmentation approaches for chronic stroke t1weighted mri data. Hum. brain mapp..

[bib0027] Kamnitsas K., Ledig C., Newcombe V.F., Simpson J.P., Kane A.D., Menon D.K., Rueckert D., Glocker B. (2017). Efficient multi-scale 3D CNN with fully connected CRF for accurate brain lesion segmentation. Med. Image Anal..

[bib0028] Karnath H.-O., Rennig J. (2017). Investigating structure and function in the healthy human brain: validity of acute versus chronic lesion-symptom mapping. Brain Struct. Funct..

[bib0029] Krizhevsky A., Sutskever I., Hinton G.E. (2012). ImageNet classification with deep convolutional neural networks. Proceedings of the Advances in Neural Information Processing Systems.

[bib0030] M. Lai, Deep learning for medical image segmentation, arXiv preprint arXiv:1505.02000, 2015.

[bib0031] LeCun Y., Bottou L., Bengio Y., Haffner P. (1998). Gradient-based learning applied to document recognition. Proc. IEEE.

[bib0032] Ledig C., Theis L., Huszár F., Caballero J., Cunningham A., Acosta A., Aitken A., Tejani A., Totz J., Wang Z. (2017). Photo-realistic single image super-resolution using a generative adversarial network. Proceedings of the IEEE Conference on Computer Vision and Pattern Recognition.

[bib0033] Liew S.-L., Anglin J.M., Banks N.W., Sondag M., Ito K.L., Kim H., Chan J., Ito J., Jung C., Khoshab N. (2018). A large, open source dataset of stroke anatomical brain images and manual lesion segmentations. Sci. Data.

[bib0034] Lyksborg M., Puonti O., Agn M., Larsen R. (2015). An ensemble of 2D convolutional neural networks for tumor segmentation. Proceedings of the Scandinavian Conference on Image Analysis.

[bib0035] Maier O., Schröder C., Forkert N.D., Martinetz T., Handels H. (2015). Classifiers for ischemic stroke lesion segmentation: a comparison study. PloS one.

[bib0036] Menze B., Jakab A., Bauer S., Kalpathy-Cramer J., Farahani K., Kirby J., Burren Y., Porz N., Slotboom J., Wiest R., Lanczi L., Gerstner E., Weber M.-A., Arbel T., Avants B., Ayache N., Buendia P., Collins L., Cordier N., Corso J., Criminisi A., Das T., Delingette H., Demiralp C., Durst C., Dojat M., Doyle S., Festa J., Forbes F., Geremia E., Glocker B., Golland P., Guo X., Hamamci A., Iftekharuddin K., Jena R., John N., Konukoglu E., Lashkari D., Antonio Mariz J., Meier R., Pereira S., Precup D., Price S.J., Riklin Raviv T., Reza S., Ryan M., Schwartz L., Shin C.-H., Shotton J., Silva C., Sousa N., Subbanna N., Szekely G., Taylor T., Thomas O., Tustison N., Unal G., Vasseur F., Wintermark M., Hye Ye D., Zhao L., Zhao B., Zikic D., Prastawa M., Reyes M., Leemput K.V. (2014). The multimodal brain tumor image segmentation benchmark (BRATS). IEEE Trans. Med. Imaging.

[bib0037] Milletari F., Navab N., Ahmadi S.A. (2016). V-net: Fully convolutional neural networks for volumetric medical image segmentation. Proceedings of the Fourth International Conference on 3D Vision (3DV).

[bib0038] Neumann A.B., Jonsdottir K.Y., Mouridsen K., Hjort N., Gyldensted C., Bizzi A., Fiehler J., Gasparotti R., Gillard J.H., Hermier M. (2009). Interrater agreement for final infarct MRI lesion delineation. Stroke.

[bib0039] Ochfeld E., Newhart M., Molitoris J., Leigh R., Cloutman L., Davis C., Crinion J., Hillis A.E. (2010). Ischemia in broca area is associated with broca aphasia more reliably in acute than in chronic stroke. Stroke.

[bib0040] Paszke A., Gross S., Chintala S., Chanan G., Yang E., DeVito Z., Lin Z., Desmaison A., Antiga L., Lerer A. (2017). Automatic differentiation in pytorch. Proceedings of the NIPS-W.

[bib0041] Pillay S.B., Stengel B.C., Humphries C., Book D.S., Binder J.R. (2014). Cerebral localization of impaired phonological retrieval during rhyme judgment. Ann. Neurol..

[bib0042] Plaut D.C., McClelland J.L., Seidenberg M.S., Patterson K. (1996). Understanding normal and impaired word reading: computational principles in quasi-regular domains. Psychol. Rev..

[bib0043] Pustina D., Coslett H.B., Turkeltaub P.E., Tustison N., Schwartz M.F., Avants B. (2016). Automated segmentation of chronic stroke lesions using linda: lesion identification with neighborhood data analysis. Hum. Brain Mapp..

[bib0044] Qi K., Yang H., Li C., Liu Z., Wang M., Liu Q., Wang S. (2019). X-net: Brain stroke lesion segmentation based on depthwise separable convolution and long-range dependencies. Proceedings of the International Conference on Medical Image Computing and Computer-Assisted Intervention.

[bib0045] Rachmadi M., Valdés-Hernández M., Agan M., Komura T. (2017). Deep learning vs. conventional machine learning: Pilot study of WMH segmentation in brain MRI with absence or mild vascular pathology. J. Imaging.

[bib0046] Ricci P.E., Burdette J.H., Elster A.D., Reboussin D.M. (1999). A comparison of fast spin-echo, fluid-attenuated inversion-recovery, and diffusion-weighted mr imaging in the first 10 days after cerebral infarction. Am. J. Neuroradiol..

[bib0047] Ronneberger O., Fischer P., Brox T. (2015). U-net: Convolutional networks for biomedical image segmentation. Proceedings of the International Conference on Medical Image Computing and Computer-Assisted Intervention.

[bib0048] S. Ruder, An overview of gradient descent optimization algorithms, arXiv preprint arXiv:1609.04747, 2016.

[bib0049] Soliman A., Khalifa F., Elnakib A., El-Ghar M.A., Dunlap N., Wang B., Gimel’farb G., Keynton R., El-Baz A. (2016). Accurate lungs segmentation on ct chest images by adaptive appearance-guided shape modeling. IEEE Trans. Med. Imaging.

[bib0050] Tseng K.-L., Lin Y.-L., Hsu W., Huang C.Y. (2017). Joint sequence learning and cross-modality convolution for 3D biomedical segmentation. Proceedings of the IEEE Conference on Computer Vision and Pattern Recognition (CVPR).

[bib0051] Tyler L.K., Marslen-Wilson W., Stamatakis E.A. (2005). Dissociating neuro-cognitive component processes: voxel-based correlational methodology. Neuropsychologia.

[bib0052] Wilcoxon F. (1945). Individual comparisons by ranking methods. Biometrics Bull..

[bib0053] Wilke M., de Haan B., Juenger H., Karnath H.O. (2011). Manual, semi-automated, and automated delineation of chronic brain lesions: A comparison of methods. NeuroImage.

[bib0054] Wong K.C., Moradi M., Tang H., Syeda-Mahmood T. (2018). 3d segmentation with exponential logarithmic loss for highly unbalanced object sizes. Proceedings of the International Conference on Medical Image Computing and Computer-Assisted Intervention.

[bib0055] Woollams A.M., Halai A., Ralph M.A.L. (2018). Mapping the intersection of language and reading: the neural bases of the primary systems hypothesis. Brain Struct. Funct..

[bib0056] Zhou, Y., Huang, W., Dong, P., Xia, Y., Wang, S., D-unet: a dimension-fusion u shape network for chronic stroke lesion segmentation, IEEE/ACM transactions on computational biology and bioinformatics, 2019. 10.1109/TCBB.2019.2939522 31502985

[bib0057] Zijdenbos A.P., Dawant B.M., Margolin R.A., Palmer A.C. (1994). Morphometric analysis of white matter lesions in mr images: method and validation. IEEE Trans. Med. Imaging.

