# Supplementary material for: A multi-path 2.5 dimensional convolutional neural network system for segmenting stroke lesions in brain MRI images
Source: Neuroimage Clin. 2019 Dec 9;25:102118. doi: 10.1016/j.nicl.2019.102118 (PMC6931186; doi:10.1016/j.nicl.2019.102118)
Supplement: Supplementary Data S1 — Supplementary Raw Research Data. This is open data under the CC BY license http://creativecommons.org/licenses/by/4.0/ [file mmc1.pdf]

Supplementary Material for "A multi-path 2.5 dimensional convolutional neural network system for segmenting stroke lesions in brain MRI images"

Yunzhe Xue[a], Fadi G. Farhat[a], Olga Boukrina[b,c], A. M. Barrett[b,c], Jeffrey R. Binder[d], Usman W. Roshan[a,*], William W. Graves[e]

[a]*Department of Computer Science, New Jersey Institute of Technology, Newark, NJ 07102, USA*
[b]*Stroke Rehabilitation Research, Kessler Foundation, West Orange, NJ, USA*
[c]*Department of Physical Medicine and Rehabilitation, Rutgers – New Jersey Medical School, Newark, NJ, USA*
[d]*Department of Neurology, Medical College of Wisconsin, Milwaukee, WI, USA*
[e]*Department of Psychology, Rutgers University – Newark, Newark, NJ, USA*

1. Shape correction

In Figure 4 of our paper we perform Stack and Squeeze shape correction operations. These are are strictly technical steps required for functions in the Pytorch deep learning library that we use to implement our model.

*Corresponding author
Email address: `usman@njit.edu` (Usman W. Roshan)

2. Multiple paths

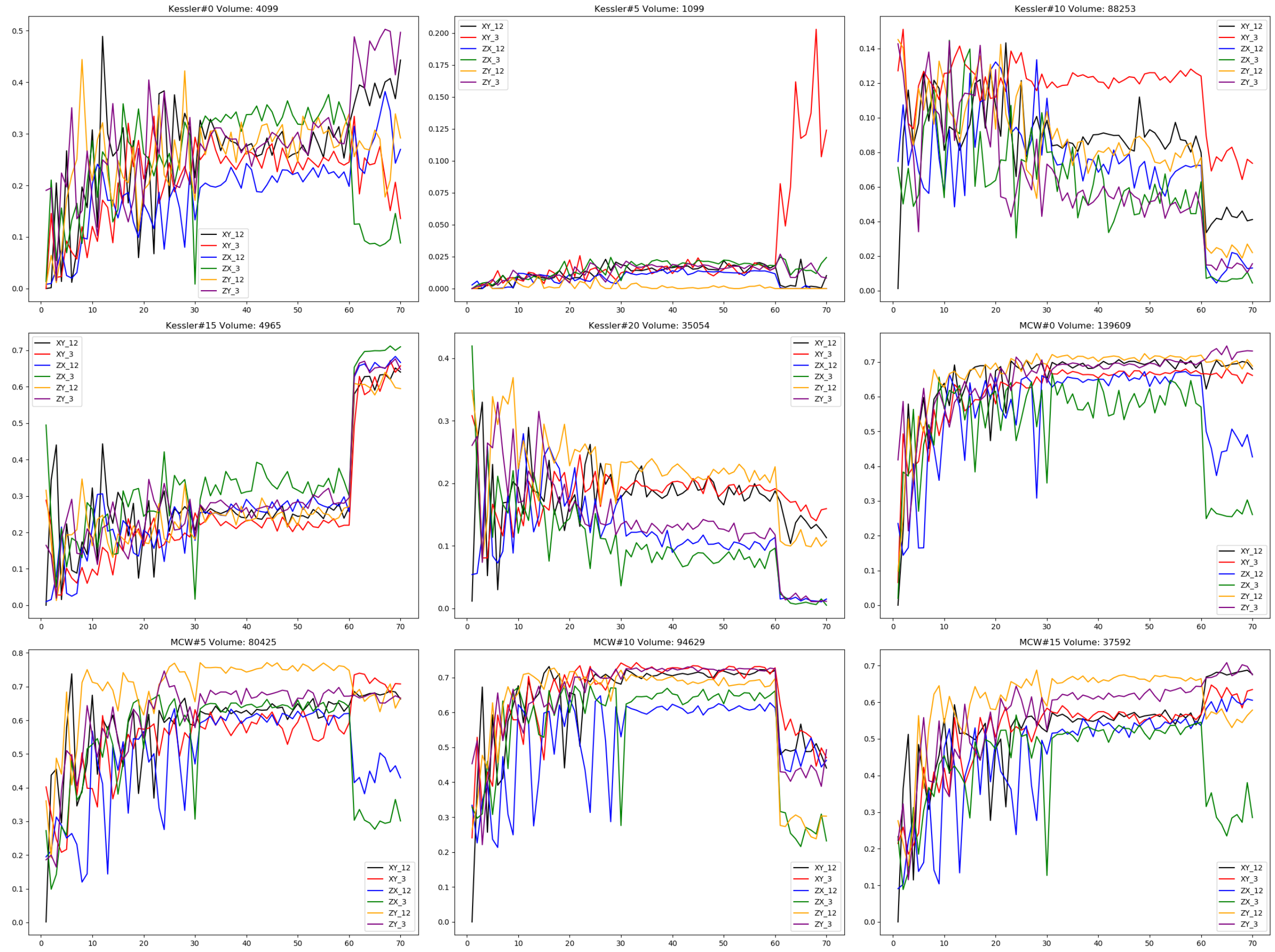

Figure 1: Test accuracy of nine samples in each of six different paths across different epochs of our model. We see no one path is consistently best.
